# Estimating the Non-Monetary Burden of Neurocysticercosis in Mexico

**DOI:** 10.1371/journal.pntd.0001521

**Published:** 2012-02-21

**Authors:** Rachana Bhattarai, Christine M. Budke, Hélène Carabin, Jefferson V. Proaño, Jose Flores-Rivera, Teresa Corona, Renata Ivanek, Karen F. Snowden, Ana Flisser

**Affiliations:** 1 Department of Veterinary Integrative Biosciences, College of Veterinary Medicine, Texas A&M University, College Station, Texas, United States of America; 2 Department of Biostatistics and Epidemiology, University of Oklahoma Health Sciences Center, Oklahoma City, Oklahoma, United States of America; 3 Hospital de Especialidades, Centro Médico Nacional Siglo XXI, Instituto Mexicano del Seguro Social, México Distrito Federal, Mexico; 4 Clinical Laboratory of Neurodegenerative Diseases, National Institute of Neurology and Neurosurgery, México Distrito Federal, Mexico; 5 Department of Veterinary Pathobiology, College of Veterinary Medicine, Texas A&M University, College Station, Texas, United States of America; 6 Facultad de Medicina, Universidad Nacional Autónoma de México (UNAM), México Distrito Federal, Mexico; Swiss Tropical Institute, Switzerland

## Abstract

**Background:**

Neurocysticercosis (NCC) is a major public health problem in many developing countries where health education, sanitation, and meat inspection infrastructure are insufficient. The condition occurs when humans ingest eggs of the pork tapeworm *Taenia solium*, which then develop into larvae in the central nervous system. Although NCC is endemic in many areas of the world and is associated with considerable socio-economic losses, the burden of NCC remains largely unknown. This study provides the first estimate of disability adjusted life years (DALYs) associated with NCC in Mexico.

**Methods:**

DALYs lost for symptomatic cases of NCC in Mexico were estimated by incorporating morbidity and mortality due to NCC-associated epilepsy, and morbidity due to NCC-associated severe chronic headaches. Latin hypercube sampling methods were employed to sample the distributions of uncertain parameters and to estimate 95% credible regions (95% CRs).

**Findings:**

In Mexico, 144,433 and 98,520 individuals are estimated to suffer from NCC-associated epilepsy and NCC-associated severe chronic headaches, respectively. A total of 25,341 (95% CR: 12,569–46,640) DALYs were estimated to be lost due to these clinical manifestations, with 0.25 (95% CR: 0.12–0.46) DALY lost per 1,000 person-years of which 90% was due to NCC-associated epilepsy.

**Conclusion:**

This is the first estimate of DALYs associated with NCC in Mexico. However, this value is likely to be underestimated since only the clinical manifestations of epilepsy and severe chronic headaches were included. In addition, due to limited country specific data, some parameters used in the analysis were based on systematic reviews of the literature or primary research from other geographic locations. Even with these limitations, our estimates suggest that healthy years of life are being lost due to NCC in Mexico.

## Author Summary

Neurocysticercosis (NCC) is a major public health problem caused by the larvae of the parasite *Taenia solium*. The condition occurs when humans ingest eggs of the pork tapeworm *Taenia solium*, which then develop into larvae in the central nervous system. The disease is predominantly found and considered important in Latin American, Asian, and African countries and is associated with a large social and economic burden. Very few studies have been conducted to evaluate the burden of NCC and there are no estimates from Mexico. We estimated the disability adjusted life years (DALYs) lost due to NCC in Mexico incorporating morbidity and mortality due to NCC-associated epilepsy, and morbidity due to NCC-associated severe chronic headaches. NCC-associated epilepsy and severe chronic headaches were estimated to cause a loss of approximately 0.25 healthy year of life per 1,000 persons annually in Mexico. This is the first estimate of DALYs associated with NCC in Mexico. However, this value is likely to be underestimated since only the clinical manifestations of epilepsy and severe chronic headaches were included.

## Introduction

Neurocysticercosis (NCC) is a major public health problem caused by the larvae of the zoonotic cestode *Taenia solium*. Humans are the definitive hosts of *T. solium* and become infected with the intestinal adult tapeworm (taeniasis) by ingesting undercooked pork containing cysticerci. Humans can also become accidental intermediate hosts after ingesting *T. solium* eggs leading to cysticercosis and/or NCC, which occurs when larvae develop in the central nervous system. A recent meta-analysis of published studies on the frequency of NCC estimated that 29% (95% CI: 23%–36%) of epilepsy cases in NCC-endemic areas exhibit NCC lesions as identified by brain neuroimaging [1]. NCC may also manifest as migraine-type headaches and stroke, among others [2], [3].

NCC is common in many developing countries where health education, sanitation, and meat inspection infrastructure are insufficient [4]. This disease is predominantly found and considered endemic in Latin American, Asian, and Sub-Saharan African countries [5], [6], [7]. In Mexico, NCC is one of the main causes of late onset epilepsy [8]. Very few studies have been conducted to evaluate the burden of NCC [9], [10] and there are no estimates from Mexico. The disability adjusted life year (DALY), developed for the Global Burden of Disease (GBD) Study, is the most common metric used to measure disease burden. It combines years of life lost due to premature mortality (YLL) and years of life lost due to time lived in a disability state (YLD). One DALY is considered the equivalent of one year of healthy life lost [11].

Two previous studies, both conducted in Africa, have evaluated the burden of cysticercosis [9], [10]. A study in Cameroon revealed that the average number of DALYs lost due to NCC was 9.0 per 1,000 person-years (95% CR: 2.8–20.4) and the monetary burden per case of cysticercosis amounted to 194 Euro (95% CR: 147–253) [10]. This estimate only accounted for the disease burden due to NCC-associated epilepsy and used serology for the diagnosis of NCC. Another study conducted in South Africa estimated that the monetary burden of NCC varied from US$ 632 to US$ 844 per NCC-associated epilepsy case, indicating high financial losses associated with this condition [9].

Studies are needed to estimate the burden of NCC in endemic countries, such as Mexico, to facilitate international comparison of disease burden and identify priorities for control. The research presented here provides the first estimate of DALYs associated with NCC in Mexico, incorporating two common clinical manifestations of patients with NCC, epilepsy and severe chronic headaches [12].

## Materials and Methods

### Study area

This study was conducted in Mexico, which is the third largest country in Latin America, with a 2005 population of almost 103 million and an annual population growth rate of 1.1% [13]. Traditional pig rearing practices in NCC endemic areas allow pigs to have access to human feces in open fields facilitating the completion of the *T. solium* life cycle [14].

### Estimation of the prevalence of epilepsy in Mexico

**Figure 1:**
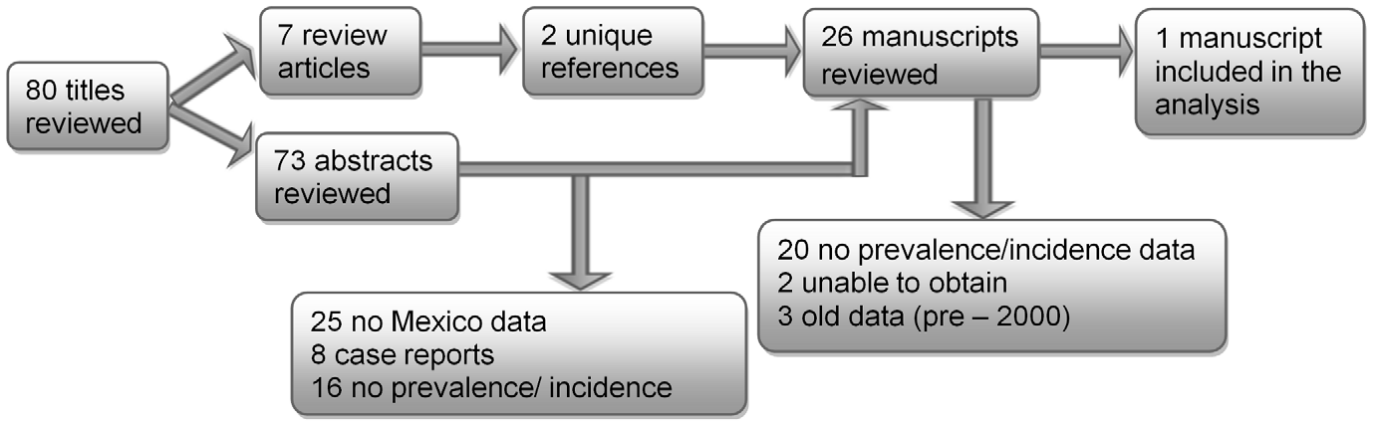
Flowchart of study selection for the literature review of epilepsy prevalence in Mexico.

Literature published through June 2011 was searched in PubMed using the key words expressions “epilepsy AND prevalence AND Mexico” and “epilepsy AND incidence AND Mexico”. Articles that were referenced in the literature identified through the original search were also obtained and reviewed (Figure 1). Community-based studies that employed door-to-door surveys of households or surveys of school children were eligible for inclusion if they were carried out by trained personnel using standardized previously validated questionnaires. In addition, data from systematic reviews and meta-analyses on epilepsy frequency were included as appropriate. The identified literature was further evaluated for epilepsy frequency data reported separately for adults and children and for urban and rural areas. For the purpose of this study, epilepsy was defined as the occurrence of at least two unprovoked seizures separated by at least 24 hours [15].

The epilepsy prevalence estimates used in the current study were based on estimates from Quet et al. (2011) [16]. This study reported epilepsy prevalence for males and females in different age groups (<15 years, 15–40 years, 41–60 years and >60 years) in a rural community in the Puebla State of Mexico in 2007. For analysis purposes, the prevalence estimates were applied to GBD age groups as appropriate. Uncertainty was modeled with uniform distributions using the upper and lower confidence intervals values from the Quet et al. (2011) study. A systematic review of published literature on epilepsy conducted in Latin America in 2005 reported that the prevalence of epilepsy in urban and rural areas was not significantly different [17]. Since there were no comparable data available specifically for Mexico, it was assumed that the prevalence of epilepsy in urban and rural areas of Mexico is also not significantly different.

### Estimation of the number of NCC cases with epilepsy in Mexico

**Figure 2:**
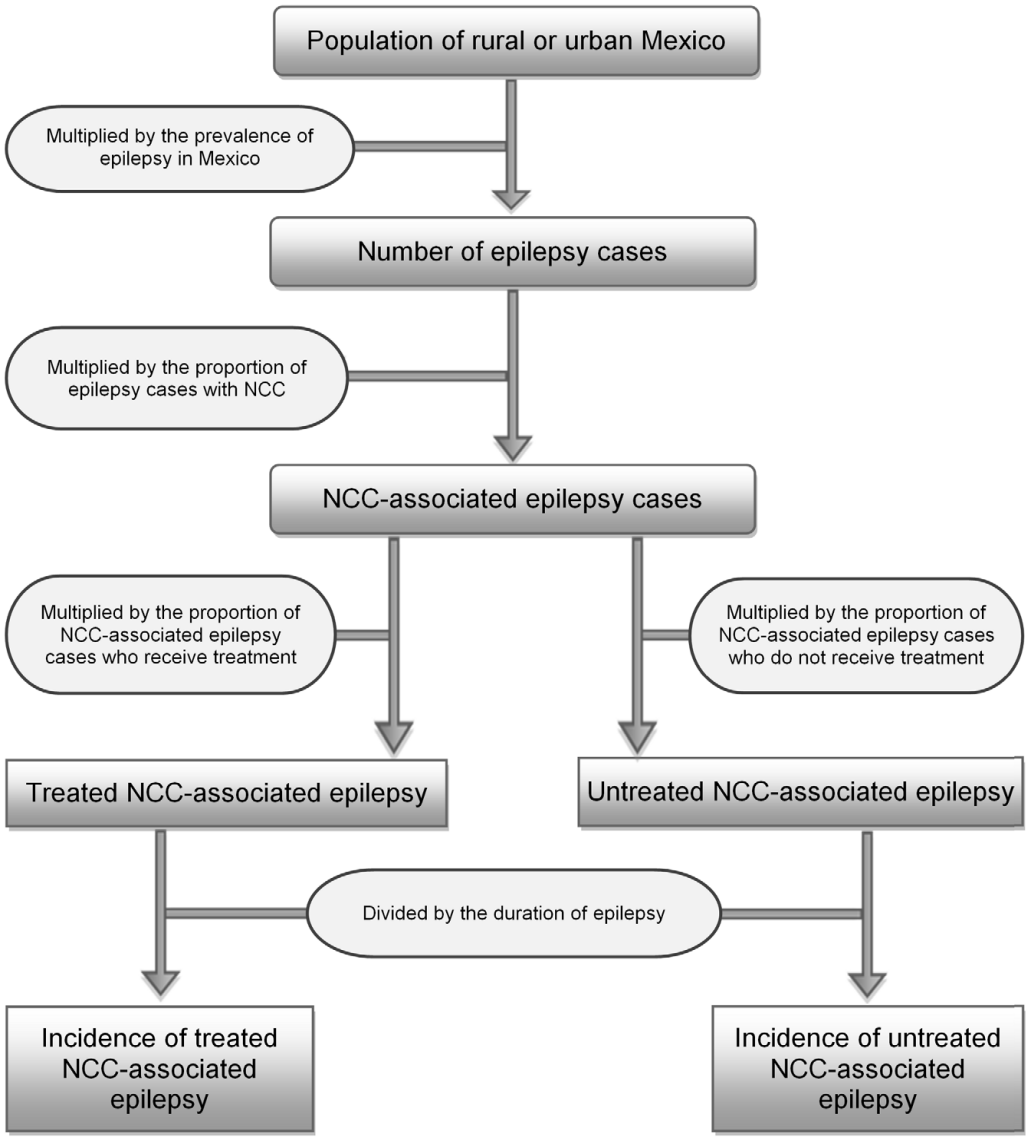
Flowchart for estimating the incidence of NCC-associated epilepsy in Mexico. Note: Please refer to Table 1 for information concerning the uncertainty distributions associated with the specific parameters. All data were stratified by rural/urban residence.

A flowchart depicting how incidence of treated and untreated NCC-associated epilepsy was determined is shown in Figure 2. The number of cases of epilepsy was estimated by multiplying the age and rural/urban stratified population size from the 2005 census [13] by the epilepsy prevalence estimates described above [16]. While the prevalence of epilepsy in rural and urban areas of Mexico was not assumed to be different, the proportion of NCC-associated epilepsy cases in urban and rural areas was assumed to be different. This assumption was based on the only identified study to look at the prevalence of NCC among patients with active epilepsy in rural versus urban clusters [18]. Although the identified study was conducted in the Vellore district of India, the difference between urban and rural areas likely reflects a difference in *T. solium* endemicity, which would also be found in Mexico. For example, pig rearing practices used in the rural areas of Mexico facilitate the completion of the *T. solium* life cycle.

The estimated proportion of epilepsy cases with NCC lesions from a meta-analysis of NCC frequency data [1] was used for rural areas of Mexico (Table 1). This value was chosen because the meta-analysis used data from primarily rural endemic communities in countries such as Bolivia, Ecuador, Honduras, Burkina Faso, and India to calculate the proportion of epilepsy cases with NCC lesions. The Vellore, India study reported the prevalence of NCC among patients with acute epilepsy in urban areas to be approximately 0.52 times that in rural areas [18]. Since such information is not available from Mexico, the lower and upper confidence bounds of the proportion of children and adults with NCC-associated epilepsy from the meta-analysis [1] were multiplied by 0.52 to obtain the corresponding lower and upper confidence bounds of the proportion of children and adults with NCC-associated epilepsy in urban areas. The estimated numbers of adults and children with epilepsy in rural and urban areas were then multiplied by the respective proportion of people with epilepsy with NCC lesions to obtain the number of NCC-associated epilepsy cases in adults and children in rural and urban areas.

**Table 1:** Epidemiological parameters used to calculate DALYs for NCC-associated epilepsy and severe chronic headaches in Mexico.

Parameter	Value or range of values	Distribution	References
2005 Population of Mexico ('000)			
Urban areas	78,986	Fixed	[13]
Rural areas	24,276	Fixed	[13]
Total	103,263	Fixed	[13]
Prevalence of epilepsy in 0–14 year old males in Mexico (per 1,000)	6.9 (1.4–12.5)	Uniform(1.4, 12.5)	[16]
Prevalence of epilepsy in 0–14 year old females in Mexico (per 1,000)	5.4 (0.8–10.0)	Uniform(0.8, 10.0)	[16]
Prevalence of epilepsy in 15–44 year old males in Mexico (per 1,000)	9.3 (1.4–17.2)	Uniform(1.4, 17.2)	[16]
Prevalence of epilepsy in 15–44 year old females in Mexico (per 1,000)	6.5 (1.4–11.7)	Uniform(1.4, 11.7)	[16]
Prevalence of epilepsy in 45–59 year old males and females in Mexico (per 1,000)	6.6 (0.1–13.2)	Uniform(0.1, 13.2)	[16]
Prevalence of epilepsy in males and females older than 60 years of age in Mexico (per 1,000)	15.5 (0.3–30.8)	Uniform(0.3, 30.8)	[16]
Proportion of epilepsy cases associated with NCC in individuals 0–14 years of age in urban areas of Mexico	0.13 (0.09–0.17)	Uniform(0.09, 0.17)	[1], [18]
Proportion of epilepsy cases associated with NCC in individuals 0–14 years of age in rural areas of Mexico	0.25 (0.18–0.32)	Uniform(0.18,0.32)	[1]
Proportion of epilepsy cases associated with NCC in individuals older than 15 years of age in urban areas of Mexico	0.15 (0.10–0.20)	Uniform (0.10,0.20)	[1], [18]
Proportion of epilepsy cases associated with NCC in individuals older than 15 years of age in rural areas of Mexico	0.28 (0.20–0.38)	Uniform (0.20,0.38)	[1]
Proportion of NCC patients 0–14 years of age with epilepsy	0.79 (0.70–0.86)	Uniform(0.70,0.86)	[22]
Proportion of NCC cases older than 15 years old with epilepsy	0.63 (0.52–0.74)	Uniform(0.52,0.74)	[22]
Proportion of epilepsy patients not receiving treatment in urban areas	0.39 (0.27–0.50)	Uniform(0.27,0.50)	[20]
Proportion of epilepsy patients not receiving treatment in rural areas	0.77 (0.68–0.87)	Uniform(0.68,0.87)	[20]
Proportion of 0–14 year-olds with severe headaches presenting with NCC	0.28 (0.21–0.35)	Uniform(0.21, 0.35)	[22]
Proportion of individuals older than 15 years of age with severe headaches presenting with NCC	0.28 (0.11–0.45)	Uniform(0.11,0.45)	[22]
Proportion of severe headaches patients not receiving treatment in urban areas	0.49 (0.37–0.60)	Uniform(0.37,0.60)	[20]
Proportion of severe headaches patients not receiving treatment in rural areas	0.87 (0.78–0.97)	Uniform(0.78,0.97)	[20]
Mortality due to epilepsy in Mexico (per 100,000 population)	1.5	Fixed	[28]

A literature review was conducted to identify information on the epilepsy treatment gap in Mexico. Literature published through June 2011 was searched in PubMed using the key words expression “epilepsy AND treatment gap AND Mexico”. When this search resulted in no usable data, a broader search using the key words expression “epilepsy AND treatment gap” was conducted. No literature was found that directly reported an estimate of the epilepsy treatment gap in Mexico. However, a 2010 systematic review of the epilepsy treatment gap literature worldwide was identified [22]. The authors of this systematic review conducted a meta-analysis of treatment gap data based on country income level (low, low middle, upper middle, and high) as defined by the World Bank [19], as well as urban versus rural location. Urban versus rural designation was based on the site description in the methods sections of the manuscripts included in the review. Based on data presented graphically in the epilepsy treatment gap meta-analysis, it was estimated that 77% (95% CI: 67%–87%) and 38% (95% CI: 27%–50%) of people with epilepsy in rural and urban areas, respectively, do not receive treatment [20]. These values were used given the unavailability of such data from Mexico. The number of NCC-associated epilepsy cases not receiving treatment was estimated by multiplying the number of NCC-associated epilepsy cases in rural and urban areas by the respective percentage not receiving treatment according to the epilepsy treatment gap meta-analysis [20]. Incidence of NCC-associated epilepsy was estimated by dividing the prevalence of NCC-associated epilepsy by the reported duration of epilepsy for different age groups from the GBD study (Table 2) [21].

**Table 2:** Disability weights and durations used to calculate NCC-associated DALYs in Mexico.

Parameter	Value or range of values	Distribution	References
Epilepsy disability weight for people between 0 and 4 years of age not receiving an appropriate treatment	0.099 (0.021–0.225)	Beta(3,27.3)	[10], [21]
Epilepsy disability weight for people between 0 and 4 years of age receiving an appropriate treatment	0.041 (0.003–0.124)	Beta(1.5,35)	[10], [21]
Epilepsy disability weight for people older than 5 years of age not receiving an appropriate treatment	0.15 (0.033–0.331)	Beta(3,17)	[10], [21]
Epilepsy disability weight for people older than 5 years of age receiving an appropriate treatment	0.065 (0.004–0.192)	Beta(1.5,21.6)	[10], [21]
Severe headaches disability weight for people not receiving an appropriate treatment	0.0275 (0.002–0.084)	Beta(1.5,53.04)	[27]
Severe headaches disability weight for people receiving an appropriate treatment	0.007 (0.000064–0.0290)	Beta(0.75,106.4)	[27]
Mean duration of disability due to epilepsy in males between 0 and 4 years of age	0.82	Fixed	[21]
Mean duration of disability due to epilepsy in males between 5 and 14 years of age	2.64	Fixed	[21]
Mean duration of disability due to epilepsy in males between 15 and 44 years of age	5.14	Fixed	[21]
Mean duration of disability due to epilepsy in males between 45 and 59 years of age	2.33	Fixed	[21]
Mean duration of disability due to epilepsy in males older than 60 years of age	1.16	Fixed	[21]
Mean duration of disability due to epilepsy in females between 0 and 4 years of age	1.27	Fixed	[21]
Mean duration of disability due to epilepsy in females between 5 and 14 years of age	3.41	Fixed	[21]
Mean duration of disability due to epilepsy in females between 15 and 44 years of age	6.83	Fixed	[21]
Mean duration of disability due to epilepsy in females between 45 and 59 years of age	6.73	Fixed	[21]
Mean duration of disability due to epilepsy in females older than 60 years of age	3.5	Fixed	[21]
Mean duration of disability due to severe headaches in males and females between 0 and 4 years of age	4.28	Fixed	[12]
Mean duration of disability due to severe headaches in males and females between 5 and 14 years of age	4.28	Fixed	[12]
Mean duration of disability due to severe headaches in males and females between 15 and 44 years of age	4.28	Fixed	[12]
Mean duration of disability due to severe headaches in males and females between 45 and 59 years of age	2.90	Fixed	[12]
Mean duration of disability due to severe headaches in males and females older than 60 years of age	4.75	Fixed	[12]

### Estimation of the total number of clinical NCC cases seen in Mexico

A recent systematic review and meta-analysis of the frequency of the main clinical manifestations associated with NCC, using documents published from January 1, 1990 to June 1, 2008, was used to estimate the proportion of children and adults with symptomatic NCC seen at neurological clinics that have epilepsy as a clinical manifestation. This meta-analysis reported that 79% (95% CI 70%–86%) of children (0–19 years old) and 63% (95% CI 52%–74%) of adults with symptomatic NCC seen in neurological clinics have epilepsy [22]. The total number of symptomatic cases of NCC receiving care in neurology clinics was calculated by dividing the number of NCC-associated epilepsy cases who seek treatment (estimated in the previous section) by the respective proportion of people with NCC that have epilepsy as a clinical manifestation. This requires the assumption that all NCC patients seeking treatment do so in neurology clinics.

### Estimation of the number of NCC cases with severe chronic headaches in Mexico

**Figure 3:**
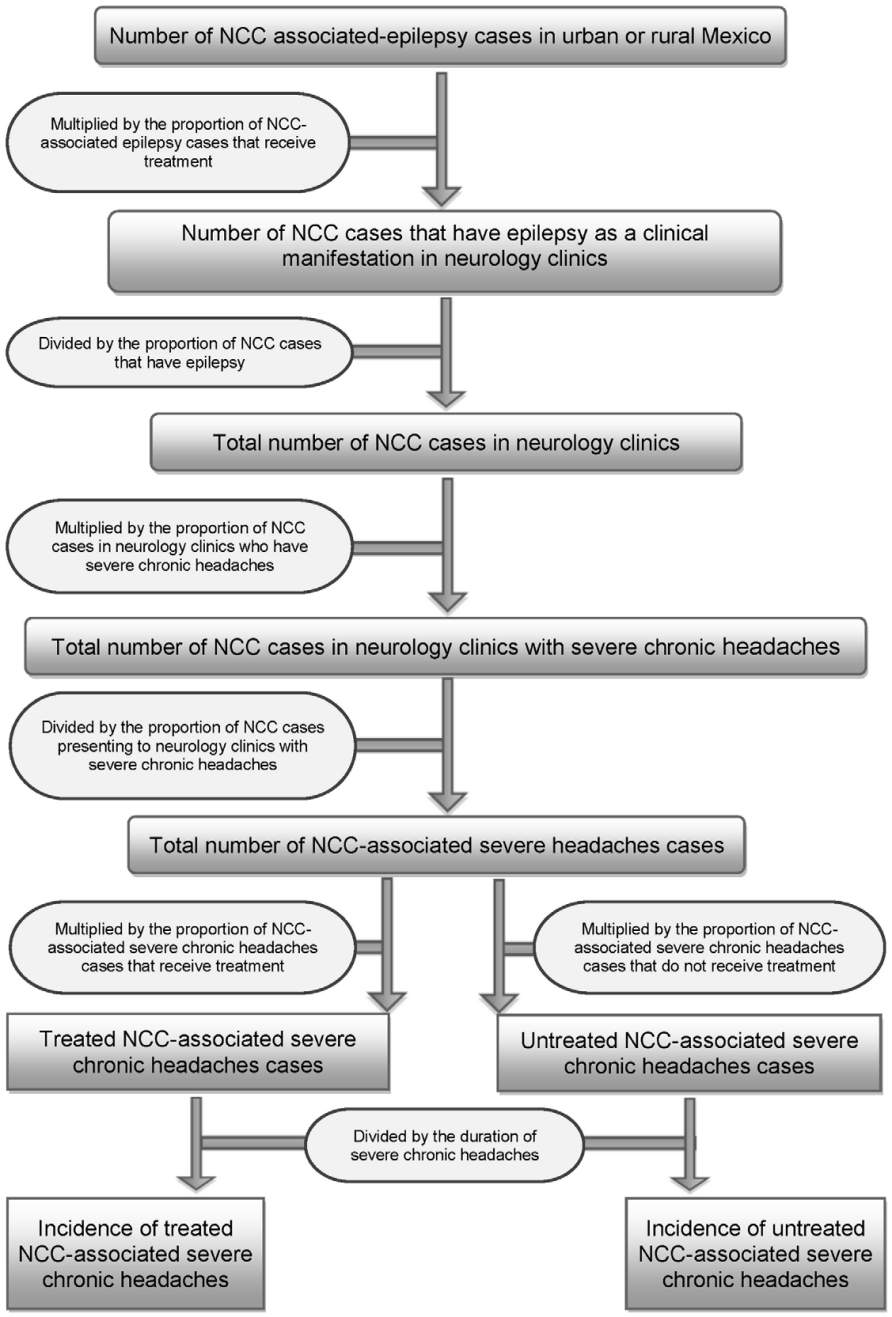
Flowchart for estimating the incidence of NCC-associated severe chronic headaches in Mexico. Note: Please refer to Table 1 for information concerning the uncertainty distributions associated with the specific parameters. All data were stratified by urban/rural residence.

A flowchart depicting how incidence of treated and untreated NCC-associated severe chronic headaches was determined is shown in Figure 3. Severe chronic headaches were defined as severe headaches that last for more than three continuous days. The number of people with NCC-associated severe chronic headaches who seek care was estimated by multiplying the total estimated rural/urban stratified numbers of NCC cases who go to neurology clinics (see previous section), by the proportion of NCC cases attending neurology clinics with headaches based on the systematic review of clinical manifestations associated with NCC [22]. The total number of people with NCC-associated severe chronic headaches in urban and rural areas was then calculated by dividing the total number of NCC-associated severe chronic headaches cases in neurological clinics by the respective proportion of NCC cases with severe chronic headaches [22].

Two scenarios were initially considered for the treatment gap for severe chronic headaches. The first scenario assumed that the epilepsy and severe chronic headaches treatment gaps were the same and the second assumed a 10% difference in treatment gaps. However, in the final analysis, the treatment gap for severe chronic headaches was assumed to be 10% higher than that of epilepsy due to the generally greater clinical severity of epilepsy. This estimate is consistent with treatment gaps reported in other countries. For example, studies conducted in the United Kingdom reported that the epilepsy treatment gap was 2%, whereas the migraine treatment gap was 14% [23], [24]. Morillo et al. (2005) estimated that 48% of the urban population of Mexico with severe headaches did not receive treatment [25]. This estimate is similar to our estimate (49%). The number of NCC-associated severe chronic headaches cases not receiving treatment in Mexico was estimated by multiplying the number of NCC-associated severe chronic headaches cases in rural and urban areas of Mexico by the respective estimated treatment gap.

The incidence of NCC-associated severe chronic headaches was estimated by dividing the prevalence estimated above by the mean duration of NCC-associated severe chronic headaches obtained from chart reviews conducted at two referral neurological hospitals in Mexico City [12]. Chart reviews captured information on duration of NCC-associated severe chronic headaches in adults 18 years of age and older from the time of diagnosis to the date when data were abstracted. The mean duration was calculated for each GBD age group (Table 2). Since chart review data were not available for children, a search of the literature was conducted to identify NCC-associated headache duration data for this age group. Literature published through June 2011 was searched in PubMed using the key words expression “neurocysticercosis AND headache AND duration”. However, no information on duration of NCC-associated headaches in children could be identified. Therefore, duration of severe chronic headaches used for the 15–44 years age group was also applied to the 0–15 years age group (Table 2).

### Parameters for the estimation of DALYs lost due to NCC-associated epilepsy and severe chronic headaches

The number of DALYs lost was calculated by adding the number of years lived with a disability (YLD) to the number of years of life lost due to mortality (YLL). The formulas used for the calculation of YLD and YLL are shown in Eqs. 1 and 2 respectively:
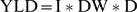
(1)where I  =  age and sex specific estimates of incidence, DW  =  disability weight, D  =  average duration of disability
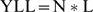
(2)where N = number of deaths per age-sex group, L = remaining life expectancy at age of death [26].

The calculation of years of life lost due to time lived in a disability state (YLD), requires the use of disability weights. Disability weights for NCC were not included in the original GBD Study or its subsequent updates. Therefore, disability weights for epilepsy provided by GBD studies were used for NCC-associated epilepsy [21]. Disability weights for severe chronic headaches were also not included in previous GBD estimates, so published disability weights for migraine were used as a surrogate [27]. Disability weights for epilepsy and severe chronic headaches used in this study are listed in Table 2.

Years of life lost due to premature mortality (YLL) were estimated using standard life expectancies (life expectancy of 82.5 years at birth for women and a life expectancy of 80.0 years at birth for men) [26]. Three percent discounting and non uniform age weighting (β = 0.04 and C = 0.1658) were applied [26]. These standard values were chosen to compare our estimates to DALYs calculated for other conditions in Mexico. World Health Organization (WHO) 2004 mortality rates for epilepsy in Mexico were used to calculate the years of life lost due to premature NCC-associated epilepsy mortality (YLL) [28]. The numbers of deaths of adults and children with epilepsy in Mexico were multiplied by the respective proportion of people with epilepsy with NCC lesions to obtain NCC-associated epilepsy deaths [1]. A review of the literature was conducted to identify data on NCC-associated headache deaths. Literature published through June 2011 was searched in PubMed using the key words expressions “neurocysticercosis AND headache AND mortality” and “neurocysticercosis AND headache AND death”. However, no publications could be identified that evaluated deaths attributable to NCC-associated headaches. Therefore, due to a lack of available data, we did not feel comfortable including an estimate for NCC-associated headaches deaths.

### Analysis

The number of DALYs lost due to NCC, with its 95% credible region (95% CR), was estimated using @Risk (Palisade Corporation, Ithaca, NY, version 4.5). Latin Hypercube sampling was used for uncertain parameters (distributions shown in Tables 1–2). The model was run for 30,000 iterations to achieve convergence. Uncertain epidemiological parameters were modeled using uniform distributions, while disability weight uncertainty was modeled using beta distributions. Regression sensitivity analysis was conducted in @ Risk by varying the value of each parameter to estimate its correlation to the total DALYs estimate. The relative values of the regression coefficients indicate which parameters had the greatest impact on the total DALYs estimate.

## Results

### Prevalence and annual incidence of NCC-associated epilepsy and severe chronic headaches cases

Approximately 0.14% of the total population of Mexico was estimated to have NCC-associated epilepsy and 0.08% was estimated to have NCC-associated severe chronic headaches. The estimated numbers of people in urban and rural areas with NCC-associated epilepsy and severe chronic headaches, along with 95% CRs, are reported in Table 3. Annual incident cases of NCC-associated epilepsy and severe chronic headaches were 0.05 and 0.02 per 100 person-years, respectively (Table 4).

**Table 3:** Estimated numbers of people with NCC-associated epilepsy and severe chronic headaches in Mexico.

NCC-associated manifestation	Area	Number	95% CR*
Epilepsy	Urban	91,355	51,079–138,347
	Rural	53,078	32,411–76,672
	All	144,433	85,230–209,162
Severe chronic headaches	Urban	47,776	17,932–99,735
	Rural	50,743	11,747–159,431
	All	98,520	34,202–231,534

*credible region.

**Table 4:** Estimated annual incidence cases of NCC-associated epilepsy and severe chronic headaches in Mexico.

NCC-associated manifestation	Area	Number	95% CR*	Incidence per 100,000 pop.
Epilepsy	Urban	30,355	17,946–43,969	29.4
	Rural	19,875	12,257–28,281	19.3
	All	50,210	30,835–70,735	48.6
Severe chronic headaches	Urban	13,069	5,288–26,295	12.7
	Rural	12,183	2,826–38,217	11.8
	All	25,253	9,310–57,537	24.5

*credible region.

### DALYs lost due to NCC-associated epilepsy and severe chronic headaches

**Figure 4:**
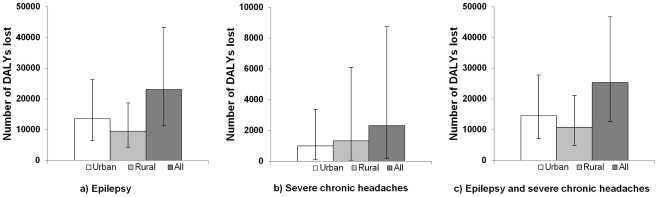
Annual number of DALYs lost due to NCC-associated epilepsy and severe chronic headaches in Mexico. (A) DALYs lost due to NCC-associated epilepsy. (B) DALYs lost due to NCC-associated severe chronic headaches. (C) DALYs lost due to NCC-associated epilepsy and severe chronic headaches. Note: The bar height in the figure represents the estimated number of DALYs lost. The plot whiskers represent the 95% CR.

The total number of DALYs lost due to NCC-associated epilepsy and severe chronic headaches in Mexico was estimated at 23,020 (95% CR: 11,283–43,276) and 2,321 (95% CR: 198–8,754), respectively, with 0.25 (95% CR: 0.12–0.46) DALY lost per 1,000 person-years (Figure 4). Twenty-eight percent of DALYs lost due to NCC was attributed to YLL and the remaining 72% was due to YLD (Table 5).

**Table 5:** Estimated annual number of DALYS due to NCC-associated epilepsy and severe chronic headaches in Mexico.

Source of DALYs	Value (DALYs)	95%CR*	Percentage of total DALYs
YLL in urban areas	4,439	3,262–5,818	18
YLL in rural areas	2,622	1,991–3,335	10
Total YLL	7,062	5,509–8,818	28
YLD in urban areas	10,143	3,063–22,920	40
YLD in rural areas	8,135	2,473–18,367	32
Total YLD	18,278	5,891–39,238	72
Total DALYs	25,341	12,569–46,640	100
DALYs per thousand persons	0.25	0.12–0.46	

*credible region.

### Sensitivity analysis

**Figure 5:**
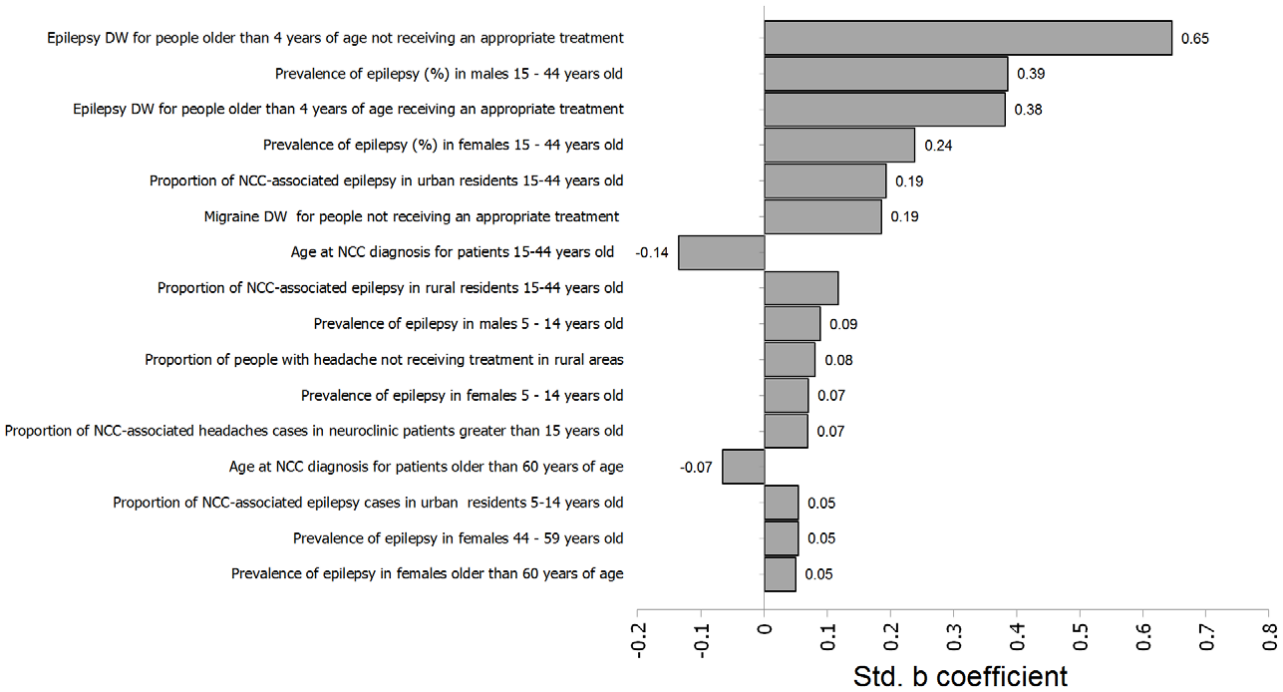
Sensitivity analysis of the total NCC-associated DALYs estimate for Mexico. Note: DW = Disability Weight.

Based on the regression sensitivity analysis, the epilepsy disability weights for untreated and for treated NCC in people older than 5 years of age and the prevalence of epilepsy in 15–44 year-old males and females were the four parameters with the greatest effect on the total DALYs estimate (Figure 5).

## Discussion

This study represents only the second study to estimate the burden of NCC using DALYs. The first, which was conducted in Cameroon, estimated human NCC burden based on epilepsy alone [10]. The estimated number of DALYs lost per 1,000 person-years was higher in Cameroon (9.0) compared to Mexico (0.25). One difference between the two studies is that all of our data were stratified by urban/rural areas, age groups, and gender. Such stratification was not used in the Cameroon study. Since the majority of the Mexican population is urban, and the proportion of epilepsy cases attributable to NCC is lower in urban areas, the overall burden per person is expected to be lower in Mexico. In addition, NCC-associated epilepsy patients were four times more likely to receive treatment in Mexico than in Cameroon. Because the disability weight for treated epilepsy is much lower than that for untreated epilepsy, this results in fewer DALYS per 1,000 person-years.

Similarly, annual number of deaths due to NCC-associated epilepsy was estimated to be higher (6.9% of the total annual incident cases) in Cameroon compared to Mexico (0.5% of the total annual incident cases). When the current model for Mexico was run using the mortality rate from the Cameroon study, 1.08 (95% CR: 0.8–1.4) DALYs per 1,000 person-years were projected to be lost compared to 0.25 (95% CR: 0.12–0.46) DALYs per 1,000 person-years. This suggests that the high mortality associated with NCC-associated epilepsy in Cameroon had a significant impact on disease burden in that country. Finally, the estimated 9 DALYs lost per 1,000 person-years [10] due to NCC-associated epilepsy in Cameroon is three times higher than the 2004 GBD estimate of 2.45 DALYs per 1,000 person-years due to all cases of epilepsy in Cameroon [28]. This suggests that the authors may have overestimated the burden of NCC associated epilepsy in that country or else that the GBD estimates for epilepsy were highly conservative.

According to 2004 GBD estimates, 1.7 DALYs per 1,000 person-years were estimated to be lost due to epilepsy in Mexico, with approximately the same number of DALYs lost due to migraine [28]. Our estimates for the number of DALYs lost per 1,000 person-years due to NCC was higher than such estimates for other helminthic infections in Mexico (ascariasis-0.05, trichuriasis-0.10, hookworm-0.03) due to the severity of clinical manifestations associated with NCC and because NCC not only causes morbidity, but also mortality in humans [28].

Our study has some limitations. The total estimated number of DALYs lost was most likely underestimated since only the NCC-associated clinical manifestations of epilepsy and severe chronic headaches were included. There are many other clinical manifestations of NCC [22] which could not be included largely due to lack of information on frequency and disability weights. Since data on the incidence of NCC-associated epilepsy and severe chronic headaches were not available, the prevalence was divided by the duration of symptoms to obtain an incidence value. In addition, we assumed that the duration of epilepsy and severe chronic headaches was the same among treated and untreated cases, which is unlikely to be accurate. The mean duration of NCC-associated severe chronic headaches was estimated from the time of diagnosis to the end of symptoms based on the review of medical charts of patients seeking care in tertiary hospitals of Mexico City [12]. This may overestimate the true duration of NCC-associated severe chronic headaches if only the most severe cases are seen in tertiary hospitals. On the other hand, it could also lead to an underestimation of the duration since people may wait a long time before seeking care. Due to limited country-specific data, parameters for the proportion of NCC patients with epilepsy and severe chronic headaches and the epilepsy treatment gap were based on systematic reviews of the literature [1], [20], [22].

Based on the regression sensitivity analysis, the disability weight used for individuals with epilepsy who were greater than 4 years of age was by far the most influential parameter. More precise values of disability weights in future versions of the GBD should reduce the uncertainty. The next most influential values were linked to the prevalence of epilepsy in Mexico. The estimates used here were based on a single study that may not fully reflect the variation of prevalence among the whole country. Better knowledge of the actual prevalence of epilepsy in Mexico would also improve our estimates.

It should be noted that DALY estimates only incorporate human health losses. However, *Taenia solium* cysticercosis not only causes losses to human health, but also to pig farmers and their communities. Therefore, the total societal burden is higher than that estimated by the number of DALYs lost. An analysis of the monetary burden of NCC in Mexico is currently underway and will be presented in a later publication.

In conclusion, this is the first estimation of the non-monetary burden of NCC in Mexico using the DALY. These estimates suggest that healthy years of life continue to be lost annually in Mexico, with a continued effort needed to control this parasitic disease in endemic regions.
